# Contrast-enhanced ultrasound for differentiating benign from malignant focal solid renal lesions in pediatric patients

**DOI:** 10.1038/s41598-024-62496-z

**Published:** 2024-05-18

**Authors:** Yusi Fu, Jia Zhong, Yan Tan, Taiqing Zheng, Minghui Liu, Guotao Wang

**Affiliations:** 1 Department of Ultrasound Diagnosis, The Second Xiang ya Hospital, Central South University, No 139, Renmin Middle Road, Changsha, 410011 Hunan People’s Republic of China; 2 Department of Ultrasound, Mawangdui District of Hunan Provincial People’s Hospital, Hunan Normal University, No 89, Guhan Road, Changsha, 410000 Hunan People’s Republic of China; 3 Department of Pathology, Hunan Children’s Hospital, No 86, Ziyuan Road, Changsha, 410007 Hunan People’s Republic of China

**Keywords:** Contrast-enhanced ultrasound, Renal lesion, Wilms tumor, Children, Cancer, Health care

## Abstract

The contrast-enhanced ultrasound (CEUS) has been mainly applied to adults to differentiate benign and malignant renal lesions, however, the characteristics of CEUS in pediatric has not been as well studied as in adults. In the present work, the eligible pediatric patients who underwent renal CEUS between March 2016 and February 2023 were retrospectively analyzed. It included 20 lesions (median diameter, 8.4 cm; range, 1.8–18.0 cm) from 20 patients (median age, 28.0 months; range, 3.0–212.0 months; 9 boys) in malignant group and 5 lesions (median diameter, 3.8 cm; range, 1.3–7.5 cm) from 5 patients (median age, 25.0 months; range, 0.7–216.0 months; 2 boys) in benign group. The diagnostic performance was assessed. Nonparametric and Chi-square tests were performed. With hyperenhancement plus wash-out, CEUS showed a sensitivity of 95.0% [95% confidence interval (CI): 75.1%, 99.9%], a specificity of 80.0% (CI: 28.4%, 99.5%), a positive predictive value of 95.0% (CI: 75.1%, 99.9%) and a negative predictive value of 80.0% (CI: 28.4%, 99.5%). It suggested that CEUS is a valuable technique for identifying between malignant and benign renal lesions in children.

## Introduction

Focal renal lesions occurring in children are relatively rare and histologically different from those affecting adults^1–3^. Wilms tumor (WT) or nephroblastoma is the most common renal malignancy in the pediatric population, accounting for about 90% of pediatric renal tumors^1,4–6^. Despite a relatively low incidence, mesoblastic nephroma is the most frequent benign solid renal tumor in neonates and infants^6^. Since the management of benign and malignant renal lesions in children is quite different, it is imperative to identify the nature of the lesions before treatment^2,7^.

Gray-scale ultrasound is considered the optimal initial examination method in children^8,9^. However, it is unrealistic to make an accurate diagnosis just by relying on gray-scale ultrasound diagnosis. For the accuracy of the diagnosis, cross-sectional imaging including contrast-enhanced computed tomography (CECT) or contrast-enhanced magnetic resonance imaging (CEMRI) imaging seems to be essential. Nevertheless, CECT and CEMRI will bring certain damage to children. For example, both CECT and CEMRI need sedation and have renal toxicity^10,11^. Especially, the CECT has radiation hazards.

Contrast-enhanced ultrasound (CEUS) is considered a useful tool for the diagnosis of a variety of abdominal diseases in children^12,13^. The intravenous applications of CEUS have been mainly applied to adults to differentiate renal lesions^10,14–16^. However, the most common pediatric CEUS use of the urethral is voiding CEUS examination with the intravesical ultrasound contrast agent injection to evaluate vesicoureteral reflux and urinary tract obstruction^17,18^. Some studies focus on the clinical use of renal intravenous CEUS in pediatric patients^19–21^. Yet, the characteristics of CEUS in pediatric focal renal lesions have not been as well studied as in adults.

The aim of this study was to analyze the CEUS characteristics of pediatric renal lesions and to differentiate benign and malignant renal lesions in children.

## Materials and methods

### Patients

**Figure 1:**
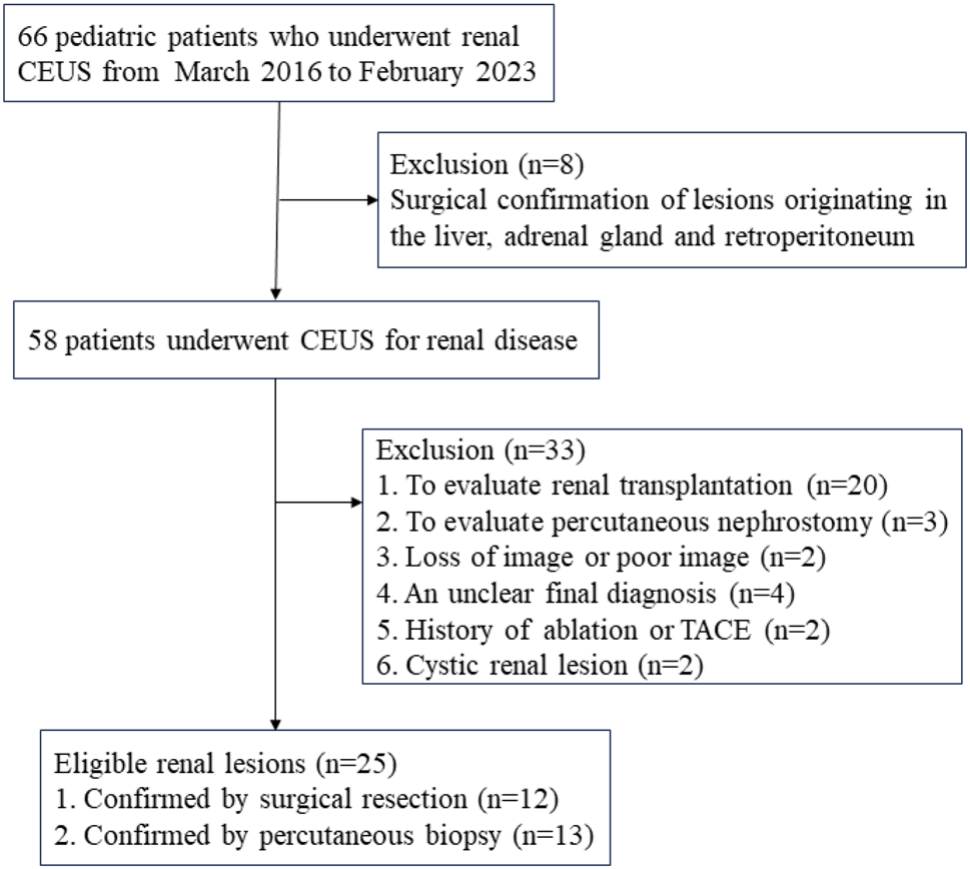
Flow diagram of the patient selection. CEUS = contrast-enhanced ultrasound.

The study was approved by the ethics committee of theSecond Xiang ya Hospital, Central South University; All research methods were performed in accordance with the relevant guidelines and regulations. Parental or legal guardians’ written informed consent was obtained. Between March 2016 and February 2023, a total of 66 pediatric patients who underwent renal CEUS examinations at the Second Xiang ya Hospital of Central South University, Changsha, China, were analyzed. At last, 41 patients were excluded (Fig. 1). The inclusion criteria were (a) age younger than 18 years old; (b) distinct focal renal lesion on gray-scale ultrasound; (c) CEUS dynamic image recording the entire enhancement pattern of the target renal lesion; (d) recognized reference standards. The exclusion criteria were (a) previously treated lesions; (b) cystic renal lesions; and (c) poor image quality. The demographic and clinical data of the pediatric patients were collected.

### Reference standard

The 25 renal lesions were confirmed by pathological examinations, with 12 patients being confirmed by surgical resection and 13 by percutaneous biopsy.

### Ultrasound examination

Ultrasound examinations were performed using a Resona 7 ultrasound system (Mindray Medical Solutions) equipped with a curvilinear transducer SC6-1 (1.0–6.0 MHz), an Aixplorer (Supersonic) equipped with a curvilinear transducer SC6-1 (1.0–6.0 MHz), or an Aplio 500 (Canon Medical Systems) equipped with a curvilinear transducer (1.9–6.0 MHz). All pediatric patients did not need to be sedated prior to examination unless an ultrasound-guided percutaneous biopsy was required. Conventional ultrasound scans were conducted before CEUS to obtain the location, number, size, internal echogenicity, and blood flow of the renal lesions. The dosage of Sono Vue (Bracco, Milan, Italy) referred to the following rules: 0.03 ml/kg, up to a maximum of 2.4 mL following the recommendation of the Food and Drug Administration. For pediatric patients under 12 months old and over, contrast agents were administered through 24-gauge and 21-gauge needles followed by 5 mL of 0.9% saline flush, respectively.

The mechanical index of the CEUS examination was less than 0.1. For patients with multiple lesions, only the largest one was selected. The target lesion was observed continuously for at least 4 min, and the entire cortical phase and parenchymal phase were stored on the hard disk. According to the 2017 version guidelines of the European Federation of Societies for Ultrasound in Medicine and Biology^22^, the renal CEUS process was classified into the cortical phase (15–30 s after contrast agent injection with cortical enhancement seen), and parenchymal phase (25 s–4 min after contrast agent injection with both cortex and medulla enhancement). If necessary, a second injection of the Sono Vue was performed 15 min after the first injection. There were no immediate and delayed adverse reactions associated with renal CEUS in children. All pediatric renal CEUS examinations were performed by a single radiologist (***, with more than 15 years of experience in renal CEUS).

### Image analysis

All conventional ultrasound and CEUS images were randomized and independently reviewed by two radiologists (***and ***, both with more than 8 years of experience in CEUS, respectively). The interval between the CEUS examination and image analysis was at least 3 months. Both readers were blinded to the final diagnosis, as well as other imaging findings of the patients. The features on CEUS of the observed lesion were recorded as follows: (a) the wash-in and wash-out pattern; (b) enhancement level in the cortical and parenchymal phase (hypo/iso/hyperenhancement). The normal renal cortex was adjacent to the solid lesion was served as the control for comparison of the enhancement. In cases of discordance, a third radiologist (***, with at least 15 years of experience in CEUS) reviewed the images to make the final decision.

### Statistical analysis

All statistical analysis was performed using SPSS software (version 22.0, IBM Corp) and MedCalc software (version 15.2.2, MedCalc Software). *P* value < 0.05 (two-tailed) was considered to indicate statistical significance. Continuous variables were presented as medians and ranges and compared by using the Mann–Whitney tests. The Chi-square tests were used for categorical variables. The diagnostic performance of CEUS in differentiating benign and malignant renal lesions was assessed. Sensitivity, specificity, positive predictive value (PPV), negative predictive value (NPV), and accuracy were calculated.

## Results

### Baseline patient characteristics

The baseline characteristics of the benign and malignant lesions group are summarized in Table 1. A total of 5 patients (median age, 25.0 months (range: 0.7–216.0 months); 2 boys) in the benign lesions group and 20 patients (median age, 28.0 months (range: 3.0 –212.0 months); 9 boys) in the malignant lesions group were further analyzed. In Table 1, the 20 malignancies were 13 WTs, 3 renal cell carcinomas (RCCs), 1 clear cell sarcoma, 1 spindle cell rhabdomyosarcoma, 1 small round cell malignancy and 1 malignant rhabdoid tumor, respectively. The 5 benign lesions were 2 mesoblastic nephromas, 1 reninoma, 1 hamartoma and 1 multicentric reticulohistiocytosis. There were no significant differences in sex, age, lesion location, and size between the two groups (all *P* > 0.05).

**Table 1:** The baseline features of 25 renal lesions in 25 patients.

	Benign lesions	Malignant lesions	*P* value
Number	5 (20.0)	20 (80.0)	
Age* (months)	25.0 (0.7–216.0)	28.0 (3.0–212.0)	0.869
Sex			1.000
Boys	2 (40.0)	9 (45.0)	
Girls	3 (60.0)	11 (55.0)	
Location			0.645
Left renal	3 (60.0)	9 (45.0)	
Right renal	2 (40.0)	11 (55.0)	
Lesion size* (cm)	3.8 (1.3–7.5)	8.4 (1.8–18.0)	0.071
Diagnosis			–
Wilms tumor	–	13 (65.0)	
Renal cell carcinoma	–	3 (15.0)	
Other malignancies	–	4 (20.0)	
Mesoblastic nephroma	2 (40.0)	–	
Other benign lesions	3 (60.0)	–	

*Data are the median, with the range in parentheses. Except where indicated, data are numbers of lesions and numbers in parentheses are percentages.

### CEUS characteristics of pediatric renal lesions

**Figure 2:**
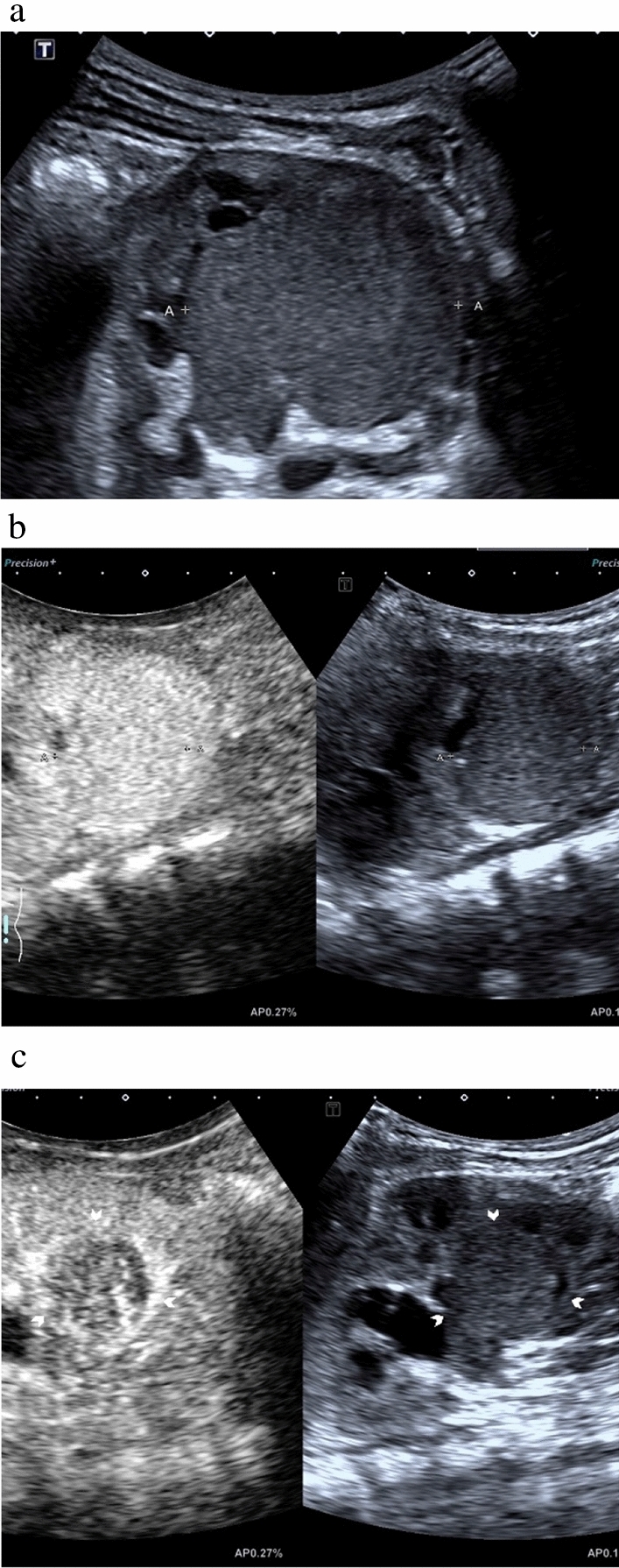
Images in a 2-year and 7-month-old boy with confirmed Wilms tumor. (**a**) Gray-scale ultrasound shows a solitary 5.5-cm homogeneous isoechoic lesion (caliber) in the left kidney. Contrast-enhanced ultrasound images of the lesion demonstrate (**b**) homogeneous isoenhancement in cortical phase (19 s) (caliber) and (**c**) wash-out in parenchymal phase (45 s) (arrowheads).

**Figure 3:**
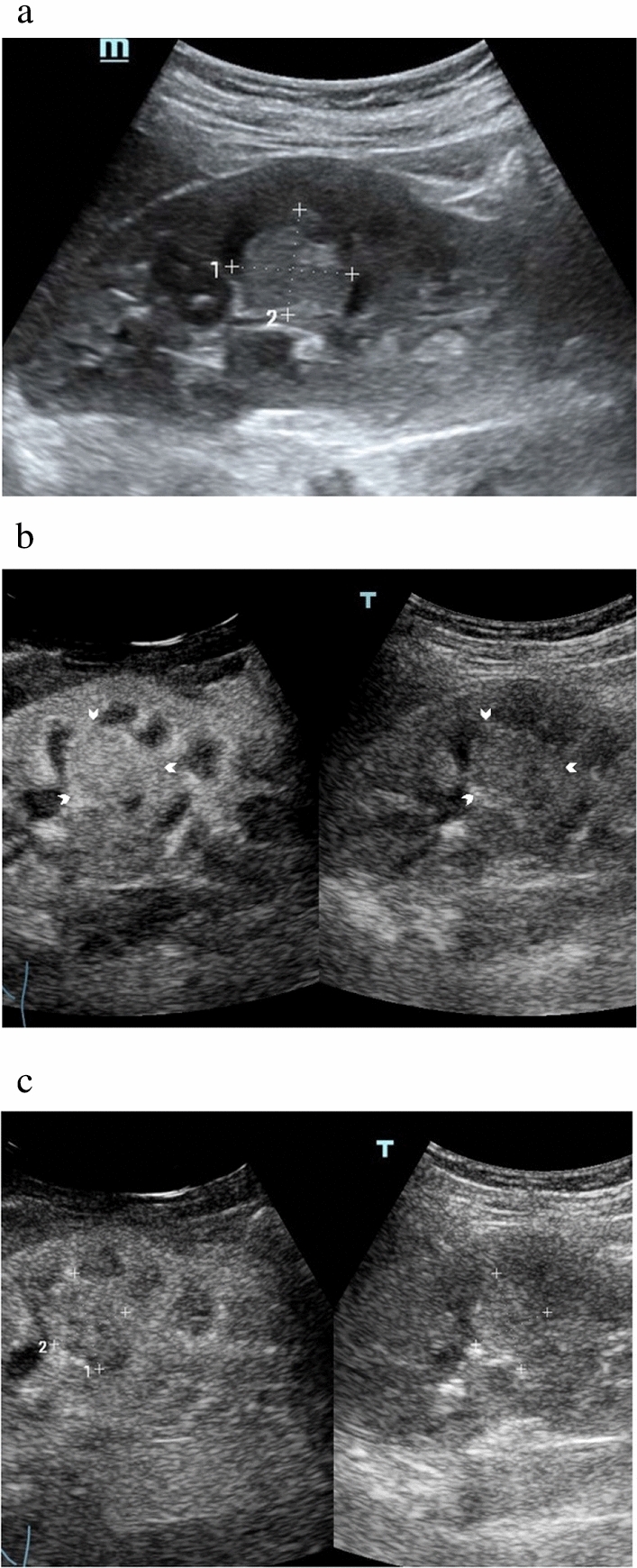
Images in a 17-year-old girl with confirmed clear cell renal cell carcinoma. (**a**) Gray-scale ultrasound shows a solitary 2.4-cm homogeneous hyperechoic lesion (calibers) in the right kidney. Contrast-enhanced ultrasound images of the lesion demonstrate (**b**) homogeneous slightly hyperenhancement in cortical phase (17 s) (arrowheads) and (**c**) isoenhancement in parenchymal phase (130 s) (calibers).

The CEUS characteristics of 25 enrolled renal lesions are summarized in Table 2. Hypo/isoenhancement was observed in 12 lesions, and hyperenhancement was observed in 13 lesions. No benign lesions were observed with hyperenhancement. Wash-out was observed in all malignancies (Fig. 2) except for a WT and two RCCs (Fig. 3). On the contrary, wash-out was not observed in benign lesions except for a reninoma.

**Table 2:** Contrast-enhanced ultrasound features of different types of renal lesions in children.

	Wilms tumor (n = 13)	Renal cell carcinoma (n = 3)	Other malignancies (n = 4)	Mesoblastic nephroma (n = 2)	Other benign lesions (n = 3)
Hypoenhancement					
No wash-out	1	0	0	0	2
Wash-out	0	0	0	0	0
Isoenhancement					
No wash-out	0	0	0	2	0
Wash-out	5	1	0	0	1
Hyperenhancement					
No wash-out	0	2	0	0	0
Wash-out	7	0	4	0	0

### Diagnostic performance of CEUS for renal benign and malignant lesions

The sensitivity, specificity, PPV, NPV, and accuracy of hyperenhancement for identification of renal malignant lesions were 65.0% (13/20), 100.0% (5/5), 100.0% (13/13), 41.7% (5/12), and 72.0% (18/25), respectively. A sensitivity of 85.0% (17 /20), specificity of 80.0% (4/5), PPV of 94.4% (17/18), NPV of 57.1% (4/7) and accuracy of 84.0% (21/25) was respectively showed when wash-out was used to distinguish renal malignant lesions. If hyperenhancement plus wash-out were used to predict renal malignant lesions, the sensitivity, specificity, PPV, NPV, and accuracy were 95.0% (19/20), 80.0% (4/5), 95.0% (19/20), 80.0% (4/5), and 92.0% (23/25), respectively (Table 3).

**Table 3:** The diagnostic performance of contrast-enhanced ultrasound in differentiating benign and malignant renal lesions in children.

	AUC	Accuracy (%)	Sensitivity (%)	Specificity (%)	PPV (%)	NPV (%)
Predictor of renal malignant lesions						
Hyperenhancement	0.825 (0.622–0.946)	72.0	65.0 (40.8–84.6)	100.0 (47.8–100.0)	100.0 (75.3–100.0)	41.7 (15.2–72.3)
Wash-out	0.825 (0.622–0.946)	84.0	85.0 (62.1–96.8)	80.0 (28.4–99.5)	94.4 (72.7–99.9)	57.1 (18.4–90.1)
Hyperenhancement or wash-out	0.875 (0.682–0.972)	92.0	95.0 (75.1–99.9)	80.0 (28.4–99.5)	95.0 (75.1–99.9)	80.0 (28.4–99.5)

AUC area under the curve, PPV positive predictive value, NPV negative predictive value.

Using CEUS, 2 renal lesions were falsely identified as malignant or benign, whereas one lesion was false positive and one lesion false negative. The false negative lesion was a WT, which occurred in the right renal was observed on ultrasound in a 3-year and 5-month-old girl. However, it showed hypoenhancement in both cortical and parenchymal phases, which was confirmed by pathological examination of surgical resection. However, the false-positive lesion was a reninoma, which was also identified by pathological examination of surgical resection in a 15-year-old girl. The lesion showed isoenhancement in the cortical phase and wash-out in the parenchymal phase.

## Discussion

In this study, we concluded the CEUS features of different types of renal lesions by analyzing 25 pediatric renal patients. By using hyperenhancement plus wash-out to predict renal malignant lesions, the sensitivity, specificity, PPV, NPV, and accuracy were 95.0%, 80.0%, 95.0%, 80.0%, and 92.0%, respectively.

WT is the most frequent tumor type for children. In our study, 92.3% (12/13) of WTs, 33.3% (1/3) of RCCs and 100.0% (4/4) of other malignancies showed hyper/isoenhancement in cortical phase and wash-out in parenchymal phase. However, the study showed that CEUS was not able to differentiate WTs from other renal malignancies in children. Since WTs rarely occur in children less than 6 months or greater than 12 years^2^, the combination of patient's age, clinical presentation, and imaging features may help diagnose WTs. One WT was misdiagnosed as a benign renal lesion because of persistent hypoenhancement in both cortical and parenchymal phases. The different enhancement patterns among WTs might be caused by the varying degrees of tumor differentiation.

CEUS is a useful method to visualize the contrast enhancement features of RCC subtypes in adults^23,24^. Clear cell RCCs show typical hyperenhancement with wash-out, while papillary RCCs and chromophobic RCCs show hypoenhancement throughout the enhancement phases^10,25^. An increasing number of studies have reported the use of renal intravenous CEUS in pediatric patients^19–21^. But the features of CEUS in pediatric RCC have not been as well studied as in adults. In the present study, a total of 3 RCCs, two clear cell RCCs and a chromophobe RCC, were included. One clear cell RCC showed isoenhancement in cortical phase and wash-out in parenchymal phase, while another clear cell RCC and the chromophobe RCC showed hyperenhancement in cortical phase without wash-out in parenchymal phase. There might be two main reasons for this. First, the sample size of our study was too small to observe the typical CEUS features of different subtypes of RCCs. Second, body circulation and biological behaviors of renal lesions between children and adults may differ. Besides, it is very important to be aware that lesion with hypoenhancement in cortical phase can be a malignancy in children. A review of pediatric kidneys also showed hypoenhancement with wash-out can be important CEUS features of renal cell carcinoma^20^.

In the present study, there were a total of 5 renal benign lesions, all of which were iso/hypoenhancement in the cortical phase. Among the 5 renal benign lesions, one reninoma showed wash-out in the parenchymal phase. The reninoma was misclassified as a malignant tumor. However, the misdiagnoses of the reninoma might be avoided due to typical clinical symptoms of hypertension and hypokalemia. Our study also showed that hyperenhancement plus wash-out was used to identify benign and malignant renal lesions in pediatric patients, with excellent diagnostic performance. These results are in line with several previous studies conducted in adults to distinguish unclear renal lesions^25–27^.

CEUS is an ideal alternative imaging for pediatric patients for the accurate characterization of unclear renal lesions, especially those with a low glomerular filtration rate, which are not suitable for CEMRI or CECT owing to renal damage, nephrogenic systemic fibrosis, or allergic reaction to iodine or gadolinium. Moreover, CEUS can be performed at the bedside, so it is suitable for pediatric patients who are intolerant to CT or MRI without sedation. As the contrast agent of CEUS is not excreted by the kidneys, the enhancement pattern of the renal lesion was not disturbed by the enhancement of the collection system. Furthermore, CEUS is a relatively inexpensive and real-time dynamic examination technique, which could be completed quickly and make the diagnosis almost simultaneously. Favorable CEUS findings with a certain diagnosis of benign lesions will greatly relieve the anxiety of parents and reduce unnecessary percutaneous biopsy and surgical resection (Supplementary information).

Our study had some limitations. First, a potential limitation of this study was its relatively small sample size because renal tumors occurring in children are relatively rare. So, it is hard to analyze the characteristics of CEUS for each tumor type. Our results need to be further validated in large sample size studies. Second, this was a retrospective single-center study in which the CEUS examinations were conducted by a single radiologist (with more than 15 years of experience in renal CEUS). For patients with multiple lesions, only the largest one was selected. Third, different imaging devices and doses of contrast agents might influence the enhancement patterns. Fourth, CEUS was not able to assess tumor staging and the overall appearance of the larger tumor. Fifth, this study did not compare the diagnostic performance between CEUS with CECT and CEMRI. Finally, renal cyst tumors were not included in this study, which may cause selection bias.

In conclusion, CEUS is a helpful tool in identifying renal benign and malignant lesions in pediatric patients.

### Supplementary Information

Supplementary Information.

## Data Availability

All data generated or analyzed during this study are included in this published article.

## Abbreviations

WT: Wilms tumor
CECT: Contrast-enhanced computed tomography
CEMRI: Contrast-enhanced magnetic resonance imaging
CEUS: Contrast-enhanced ultrasound
PPV: Positive predictive value
NPV: Negative predictive value
RCC: Renal cell carcinoma

## References

[1] Royer-Pokora B (2013). Genetics of pediatric renal tumors. Pediatr. Nephrol. (Berlin, Germany).

[2] Miniati D (2008). Imaging accuracy and incidence of Wilms’ and non-Wilms’ renal tumors in children. J. Pediatr. Surg..

[3] Chung EM, Graeber AR, Conran RM (2016). Renal tumors of childhood: Radiologic-pathologic correlation part 1. The 1st decade: From the radiologic pathology archives. Radiographics.

[4] Treger TD, Chowdhury T, Pritchard-Jones K, Behjati S (2019). The genetic changes of Wilms tumour. Nat. Rev. Nephrol..

[5] Vujanić GM (2018). The UMBRELLA SIOP-RTSG 2016 Wilms tumour pathology and molecular biology protocol. Nat. Rev. Urol..

[6] van den Heuvel-Eibrink MM (2008). Characteristics and survival of 750 children diagnosed with a renal tumor in the first seven months of life: A collaborative study by the SIOP/GPOH/SFOP, NWTSG, and UKCCSG Wilms tumor study groups. Pediatr. Blood Cancer.

[7] Servaes SE, Hoffer FA, Smith EA, Khanna G (2019). Imaging of Wilms tumor: An update. Pediatr. Radiol..

[8] Gee MS, Bittman M, Epelman M, Vargas SO, Lee EY (2013). Magnetic resonance imaging of the pediatric kidney: Benign and malignant masses. Magn. Resonance Imaging Clin. N. Am..

[9] Lee EY (2007). CT imaging of mass-like renal lesions in children. Pediatr. Radiol..

[10] Kazmierski B, Deurdulian C, Tchelepi H, Grant EG (2018). Applications of contrast-enhanced ultrasound in the kidney. Abdom. Radiol. (New York).

[11] Darge K (2013). Safety of contrast-enhanced ultrasound in children for non-cardiac applications: A review by the society for pediatric radiology (SPR) and the international contrast ultrasound society (ICUS). Pediatr. Radiol..

[12] Mao M (2019). The safety and effectiveness of intravenous contrast-enhanced sonography in chinese children-a single center and prospective study in China. Front. Pharmacol..

[13] Sidhu PS (2017). Role of contrast-enhanced ultrasound (CEUS) in paediatric practice: An EFSUMB position statement. Ultraschall Med..

[14] Herms E (2023). Ultrasound-based “CEUS-Bosniak”classification for cystic renal lesions: An 8-year clinical experience. World J. Urol..

[15] Sun D (2020). Differential diagnosis of <3 cm renal tumors by ultrasonography: A rapid, quantitative, elastography self-corrected contrast-enhanced ultrasound imaging mode beyond screening. Br. J. Radiol..

[16] Ling W (2017). Ultrasonographic findings of renal cell carcinomas associated with Xp11.2 translocation/TFE3 gene fusion. Contrast Media Mol. Imaging.

[17] Chua ME (2019). The evaluation of vesicoureteral reflux among children using contrast-enhanced ultrasound: A literature review. J. Pediatr. Urol..

[18] Yousefifard M (2022). Contrast-enhanced voiding urosonography, a possible candidate for the diagnosis of vesicoureteral reflux in children and adolescents; A systematic review and meta-analysis. J. Pediatr. Urol..

[19] Chan JP (2021). Utility of contrast-enhanced ultrasound for solid mass surveillance and characterization in children with tuberous sclerosis complex: An initial experience. Pediatr. Nephrol. (Berlin, Germany).

[20] Back SJ (2021). Contrast-enhanced ultrasound of the kidneys and adrenals in children. Pediatr. Radiol..

[21] Kapur J, Oscar H (2015). Contrast-enhanced ultrasound of kidneys in children with renal failure. J. Med. Ultrasound.

[22] Sidhu PS (2018). The EFSUMB guidelines and recommendations for the clinical practice of contrast-enhanced ultrasound (CEUS) in non-hepatic applications: Update 2017 (Short Version). Ultraschall Med..

[23] Mueller-Peltzer K, Negrao de Figueiredo G, Graf T, Rübenthaler J, Clevert DA (2019). Papillary renal cell carcinoma in contrast-enhanced ultrasound (CEUS)—a diagnostic performance study. Clin. Hemorheol. Microcirc..

[24] Dipinto P (2024). Qualitative and quantitative characteristics of CEUS for renal cell carcinoma and angiomyolipoma: A narrative review. J. Ultrasound.

[25] Rübenthaler J, Negrão de Figueiredo G, Mueller-Peltzer K, Clevert DA (2018). Evaluation of renal lesions using contrast-enhanced ultrasound (CEUS); a 10-year retrospective European single-centre analysis. Eur. Radiol..

[26] Marschner CA (2020). Comparison of computed tomography (CT), magnetic resonance imaging (MRI) and contrast-enhanced ultrasound (CEUS) in the evaluation of unclear renal lesions. RoFo: Fortschritte auf dem Gebiete der Rontgenstrahlen und der Nuklearmedizin.

[27] Rübenthaler J (2016). Comparison of magnetic resonance imaging (MRI) and contrast-enhanced ultrasound (CEUS) in the evaluation of unclear solid renal lesions. Clin. Hemorheol. Microcirc..

